# Virome and metagenomic analysis reveal the distinct distribution of microbiota in human fetal gut during gestation

**DOI:** 10.3389/fimmu.2022.1079294

**Published:** 2023-01-05

**Authors:** Xu Liu, Guolin He, Yue Lan, Weijie Guo, Xuyuan Liu, Jing Li, Anqing Liu, Miao He, Xinhui Liu, Zhenxin Fan, Yaoyao Zhang

**Affiliations:** ^1^ Key Laboratory of Bioresources and Ecoenvironment, Ministry of Education, College of Life Sciences, Department of Gynecology and Obstetrics, West China Second University Hospital, Sichuan University, Chengdu, China; ^2^ Sichuan Key Laboratory of Conservation Biology on Endangered Wildlife, College of Life Sciences, Sichuan University, Chengdu, China; ^3^ Key Laboratory of Birth Defects and Related Diseases of Women and Children of Ministry of Education, Department of Gynecology and Obstetrics, West China Second University Hospital, Sichuan University, Chengdu, China; ^4^ Institute of Blood Transfusion, Chinese Academy of Medical Sciences, Chengdu, Sichuan, China

**Keywords:** fetal gut microbiota, metagenomics, virome, archaea, immunity, gestation

## Abstract

Studies have shown that fetal immune cell activation may result from potential exposure to microbes, although the presence of microbes in fetus has been a controversial topic. Here, we combined metagenomic and virome techniques to investigate the presence of bacteria and viruses in fetal tissues (small intestine, cecum, and rectum). We found that the fetal gut is not a sterile environment and has a low abundance but metabolically rich microbiome. Specifically, Proteobacteria and Actinobacteria were the dominant bacteria phyla of fetal gut. In total, 700 species viruses were detected, and *Human betaherpesvirus 5* was the most abundant eukaryotic viruses. Especially, we first identified *Methanobrevibacter smithii* in fetal gut. Through the comparison with adults’ gut microbiota we found that Firmicutes and Bacteroidetes gradually became the main force of gut microbiota during the process of growth and development. Interestingly, 6 antibiotic resistance genes were shared by the fetus and adults. Our results indicate the presence of microbes in the fetal gut and demonstrate the diversity of bacteria, archaea and viruses, which provide support for the studies related to early fetal immunity. This study further explores the specific composition of viruses in the fetal gut and the similarities between fetal and adults’ gut microbiota, which is valuable for understanding human fetal immunity development during gestation.

## Introduction

Immune system is crucial to recognize and exclude antigenic foreign particles and maintain the stability of the internal environment. The human immune system begins to develop early in fetal development and has obvious sensitivity to external antigens (1–3). The fetal immunity is likely influenced by fragments and metabolites of maternal gut microbes, whereas the presence of the microbiome *in utero* has been a controversial topic (4). Earlier reports have shown that the amniotic cavity and placenta are sterile (5–10). The superior defenses of placenta mean it’s extremely difficult for microbes to enter the uterine environment.

It is widely known that the placenta is an essential organ for material exchange and the primary barrier between the mother and the fetus during human pregnancy. The placenta composed of amnion, villous trees and decidua basalis, has the function of defense, synthesis and immunity. Anchoring villi, an integral part of the villous tree, are attached to decidua basalis by extravillous trophoblasts (EVTs). Fetal blood passes through the umbilical artery to the villous capillaries and exchanges material with maternal blood in the intervillous space, but fetal blood and maternal blood are not directly connected. The villous trees of placenta at full term are covered by syncytiotrophoblast, and there is a layer of cytotrophoblasts below which is discontinuous. The inner layer of cytotrophoblasts layer is the basement membrane, which acts as the placental barrier (11). In addition, syncytiotrophoblasts and cytotrophoblasts both provide effective protection against viral and non-viral pathogens. Among them, the surface of syncytiotrophoblast has unique physical properties, and the physical barrier formed by syncytiotrophoblast limits the vertical transmission of pathogens at multiple stages of pregnancy (12–16).

However, with the development of microbial detection technology, more and more evidence suggest the presence of microbes in human placenta and fetus (17–26). Studies supporting the sterile womb hypothesis suggest that the microbial signals detected in the womb are actually due to contamination of samples and the DNA purification kits (21, 27). Researches supporting the presence of a low biomass placental microbiome suggest that after filtering out contaminants and low-quality sequences according to negative controls, some microbial signals still exist (26, 28, 29). Excitingly, a recent study showed that microbial exposure reduces fetal immune cells early in human development. This study demonstrated the presence of microbes in fetal organs by inoculating fetal tissue in culture media and visualizing fetal guts, and suggested that these bacteria induce the activation of syngeneic memory T cells in fetal mLN T cells. And the question of how do microbes get into the uterine environment, has it been shown that pathogens (Zika virus, Toxoplasma gondii, HIV, Cytomegalovirus, etc.) might target multiple cells in the decidua to reach the extravillous trophoblasts (EVTs) layer and eventually bypass the syncytial layer (13, 30, 31). Microbes might use this mechanism to breach the placental barrier, but are more likely to evolve distinct strategies at different stages of pregnancy (11).

Most of the recent studies on fetal microbes are based on 16S rRNA gene amplicon sequencing and metagenomic sequencing (10, 26, 32, 33). Metagenomic data includes bacterial, archaear, protozoa, virus, fungus and host genomes. Compared to 16S rRNA sequencing, the DNA used for metagenomic sequencing is not amplified by PCR, and the metagenomic results are relatively unbiased. Besides, the composition, abundance and function of microbiota can be obtained by metagenomic sequencing (34). However, due to the low biomass of fetal samples, it is difficult to detect archaea and virus signals using 16S rRNA and metagenomic sequencing. Virome is a combination of metagenomic theory and existing virus molecular biological detection technology, mainly used for the studies of all viruses genetic material in the environment (35). Therefore, in order to detect as many microbial signals as possible, virome sequencing were used in this study to explore the fetal gut microbiome based on the metagenomic results.

## Materials and methods

### Sample collection

**Figure 1:**
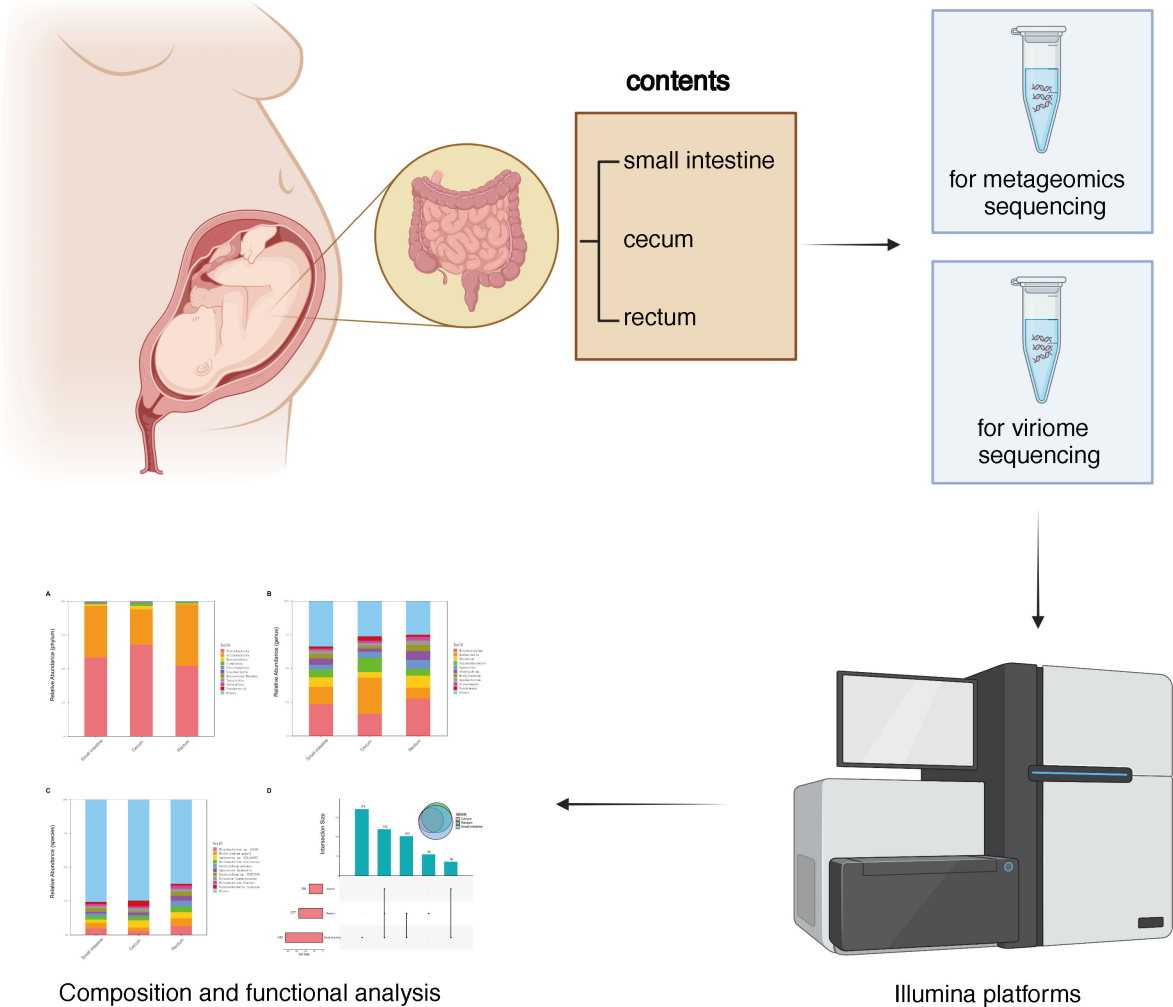
Graphical scheme and flow chart of experiment design.

Human fetal tissues were obtained in accordance from West China 2^nd^ University Hospital with ethic approval of Ethics Committees of West China 2^nd^ University Hospital. All women gave written consent to the use of fetal tissues according to internationally recognized guidelines (36). All fetal tissues (gut) were obtained from 2nd trimester (12-22 weeks) elective pregnancy terminations. The fetus was considered structurally normal on ultrasound examination prior to termination and by gross morphological examination following termination. Fetal tissues from 2nd trimester of gestation were used for this study. The participant (or mother, in the case of fetal samples) gave written informed consent. Mid-trimester terminations were medically induced and the fetus was delivered through the birth canal. Fetal organs were collected under sterile conditions in a tissue culture hood. Aseptic equipment was used for collecting the intestinal contents of fetal small intestine, cecum and rectum. The main experimental route was shown in **Figure 1**.

### Metagenomic sequencing

Fetal samples were sent to Chengdu Life Baseline Technology Co., Ltd. for metagenomic sequencing. The DNA samples were extracted using Tiangen DNA Stool Mini Kit (TIANGEN Biotech Co., Ltd. China) with the manufacturer’s instructions. In total, 0.2 μg DNA per sample was used for the DNA library preparations after DNA extraction. Sequencing library was generated using NEBNext^®^ UltraTM DNA Library Prep Kit for Illumina (NEB, USA, Catalog #: E7370L). The assessment of library quality and quantity was performed by Agilent 5400 system(Agilent, USA) and QPCR (1.5 nM), respectively. The qualified libraries were sequenced on Illumina NovaSeq 6000 with pair-end 150bp reads.

### Virome sequencing

Virome sequencing was performed in Chengdu Life Baseline Technology Co., Ltd. To remove debris and cells, samples were centrifuged at 2,500 x g for 5 minutes, and supernate was passed through a 0.45 μm filter after another centrifugation (5,000 x g, 20 min). After the treatment with 2 ul lysozyme (50 mg/ml) at 37 °C for 30 minutes, samples were treated with 0.2x volume chloroform at RT for 10 minutes. Then 10U Tubro DNase I (Ambion), 2 ug RNase A (Roche) or 20 U of RNase I (ThermoFisher Scientific) were added to the new centrifugation supernate (17,000 x g, 10 min) followed by heat inactivation at 65 °C for 10 minutes. VLPs DNA extraction and quantification were performed by Qiagen MinElute virus kit and Qubit dsDNA HS Assay Kit (ThermoFisher Scientific), respectively. After the library preparation, sequencing was performed on an Illumina Nova Seq 6000 platform using pair-end 150bp reads.

### Data analyses

To compare the gut microbiota of the fetus and adults, we downloaded 13 gut metagenomic data of healthy adults from public database (https://www.ncbi.nlm.nih.gov/). The raw data obtained from metagenomic and virome sequencing was used for subsequent analysis. Trimmomatic was used to remove the adapters and low-quality reads of raw reads after the sequencing with the setting of average quality per base >20 and minimum length 90 bp (37). The host contamination was removed by Bowtie2 with human reference genome (38). MEGAHIT (39) was used to the *de novo* assembly (–min-contig-len 300). We performed gene prediction and translation of amino acid sequences by Prodigal (40) and DIAMOND (41), respectively. The taxonomic annotation were assigned by Kraken2 (42) with option “–use-mpa-style”. Functional annotations, including microbial metabolic pathway and ARGs, were assessed by using HUMANn3 (43) and comprehensive antibiotic resistance database (CARD) (44).

## Results

### Microbial composition identified by metagenomic analysis

**Figure 2:**
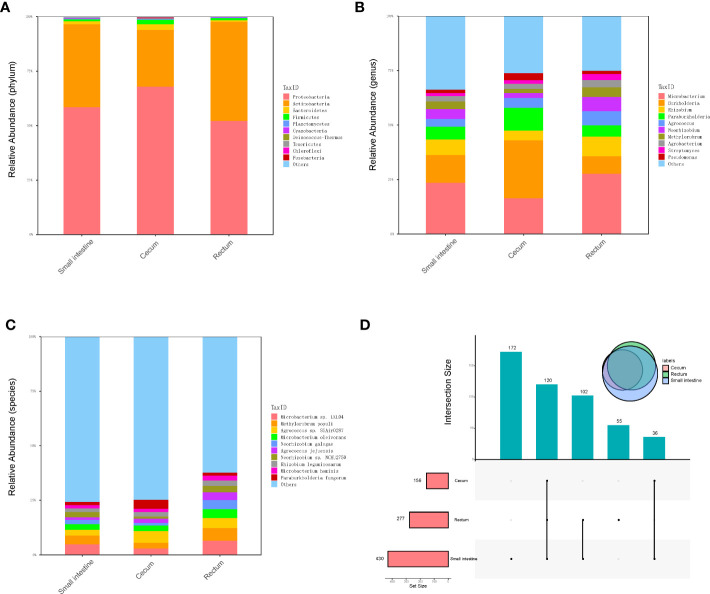
Metagenomics analysis of gut microbiota. **(A)** The top 10 abundant phyla in three different parts of fetal gut. **(B)** The top 10 abundant genera in three different parts of fetal gut. **(C)** The top 10 abundant species in three different parts of fetal gut. **(D)** Upset plot and venn plot of fetal gut microbiota in species level. The column above indicates the intersection of several samples in the row of which the point is in.

Metagenomic sequencing was performed on the contents of the fetal small intestine, cecum and rectum, a total of 486 species of bacteria were detected, including 8 phyla and 238 genera (**Figure 2A–C**). Among the 486 species, 120 species were shared by small intestine, cecum and rectum, 172 species were the peculiar species of small intestine, and the number of peculiar species in rectum was 55 (**Figure 2D**). As shown in **Table 1**, the top 3 phyla in the small intestine and cecum were Proteobacteria, Actinobacteria and Bacteroidetes. However, Firmicutes replaced Bacteroidetes as the third phylum in the rectum. At the genus level, the top 3 of the small intestine (specifically *Microbacterium*, *Burkholderia* and *Rhizobium*) and rectum (specifically *Microbacterium*, *Rhizobium* and *Burkholderia*) were similar. While there were significant changes in cecum, the top 3 genera of cecum were *Burkholderia*, *Microbacterium* and *Paraburkholderia*. At the specie level, the top 3 in relative abundances of the small intestine and rectum were *Microbacterium* sp. LKL04, *Methylorubrum populi* and *Agrococcus* sp. SGAir0287, while the top 3 species of cecum were *Agrococcus* sp. SGAir0287, *Paraburkholderia fungorum* and *Burkholderia multivorans*. In addition, very tiny amounts of viruses and archaea were detected. To further explore the function of fetal gut microbiome, we performed the functional enrichment analysis by HUMANn3, and there was no pathway enriched.

**Table 1:** Top 5 bacteria at phylum, genus and species level (metagenomics).

Level	Small intestine	Cecum	Rectum	Adult gut
Phylum	Proteobacteria	Proteobacteria	Proteobacteria	Firmicutes
	Actinobacteria	Actinobacteria	Actinobacteria	Bacteroidetes
	Bacteroidetes	Bacteroidetes	Firmicutes	Actinobacteria
	Firmicutes	Firmicutes	Bacteroidetes	Proteobacteria
	Cyanobacteria	Planctomycetes	Planctomycetes	Verrucomicrobia
Genus	*Microbacterium*	*Burkholderia*	*Microbacterium*	*Bacteroides*
	*Burkholderia*	*Microbacterium*	*Rhizobium*	*Phocaeicola*
	*Rhizobium*	*Paraburkholderia*	*Burkholderia*	*Faecalibacterium*
	*Paraburkholderia*	*Agrococcus*	*Neorhizobium*	*Bifidobacterium*
	*Neorhizobium*	*Rhizobium*	*Agrococcus*	*Roseburia*
Species	*Microbacterium sp. LKL04*	*Agrococcus sp. SGAir0287*	*Microbacterium sp. LKL04*	*Phocaeicola vulgatus*
	*Methylorubrum populi*	*Paraburkholderia fungorum*	*Methylorubrum populi*	*Faecalibacterium prausnitzii*
	*Agrococcus sp. SGAir0287*	*Burkholderia multivorans*	*Agrococcus sp. SGAir0287*	*Bacteroides uniformis*
	*Microbacterium oleivorans*	*Microbacterium sp. LKL04*	*Neorhizobium galegae*	*Roseburia intestinalis*
	*Neorhizobium sp. NCHU2750*	*Methylorubrum populi*	*Microbacterium oleivorans*	*Bifidobacterium longum*

### Microbial composition identified by virome analysis

**Figure 3:**
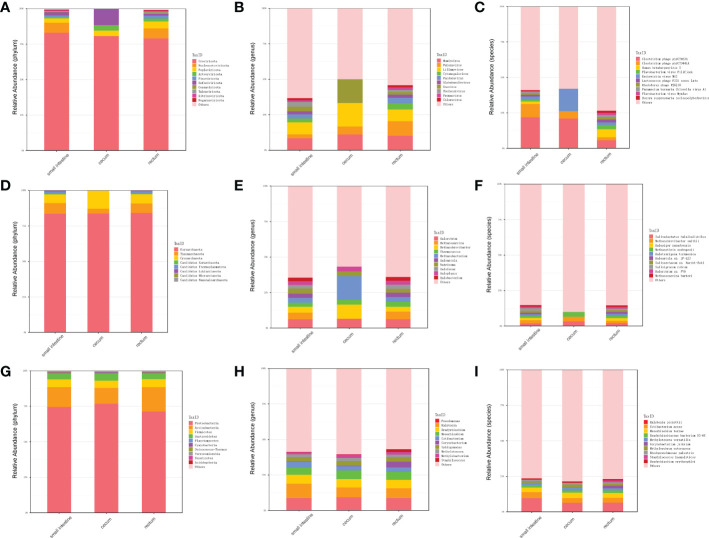
Distribution of microbiota in different parts of fetal gut detected by virome. **(A)** The top 10 abundant virus phyla in three different parts of fetal gut. **(B)** The top 10 abundant virus genera in three different parts of fetal gut. **(C)** The top 10 abundant virus species in three different parts of fetal gut. **(D)** The top 10 abundant archaea phyla in three different parts of fetal gut. **(E)** The top 10 abundant archaea genera in three different parts of fetal gut. **(F)** The top 10 abundant archaea species in three different parts of fetal gut. **(G)** The top 10 abundant bacteria phyla in three different parts of fetal gut. **(H)** The top 10 abundant bacteria genera in three different parts of fetal gut. **(I)** The top 10 abundant bacteria species in three different parts of fetal gut.

In order to explore the presence of virus in fetal gut and detect as many other microbes as possible, we performed virome sequencing and relaxed the filtering conditions of the virus sequence. Compared with metagenomic data, more viruses, bacteria and archaea were detected. In total, 700 species viruses (including 14 phyla and 432 genera, **Figure 3A–C**), 267 species of archaea (including 8 phyla and 118 genera, **Figure 3D–F**) and 5,477 species of bacteria (including 40 phyla and 1,475 genera) were detected (**Figure 3G–I**).

**Figure 4:**
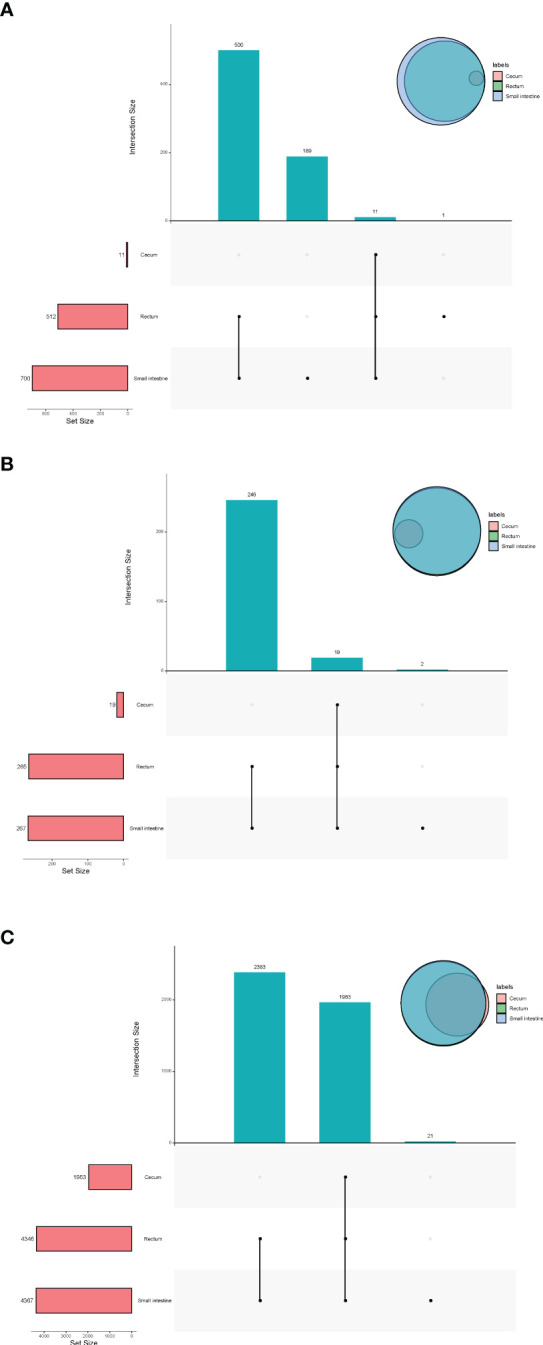
Upset plot and venn plot of fetal gut microbiota detected by virome (species level). **(A)** Upset plot and venn plot of fetal gut virus in three different parts. **(B)** Upset plot and venn plot of fetal gut archaea in three different parts. **(C)** Upset plot and venn plot of fetal gut bacteria in three different parts.

In terms of viruses, 11 species were shared by small intestine, cecum and rectum, 189 species were the peculiar species of small intestine, and the number of peculiar species in rectum was 1 (**Figure 4A**). The top 3 phyla of small intestine and rectum were Uroviricota, Nucleocytoviricota and Peploviricota, while only Uroviricota was detected in cecum. The top 3 genera of small intestine were *Lillamyvirus*, *Muminvirus* and *Inovirus*, while *Pahexavirus*, *Muminvirus* and *Lillamyvirus* were the top 3 genera of rectum. And no viral genera were detected in the cecum, which is consistent with species level. At the specie level, *Clostridium* phage phiCT453A was the most abundant species in small intestine and rectum, and *Human betaherpesvirus 5* was detected in the rectum (**Table 2**).

**Table 2:** Top 5 viruses at phylum, genus and species level (virome).

Level	Small intestine	Cecum	Rectum	Adult gut
Phylum	Uroviricota	Uroviricota	Uroviricota	Uroviricota
	Nucleocytoviricota	Hofneiviricota	Nucleocytoviricota	Hofneiviricota
	Peploviricota	Peploviricota	Peploviricota	Nucleocytoviricota
	Hofneiviricota	Artverviricota	Artverviricota	Pisuviricota
	Artverviricota		Pisuviricota	
Genus	*Lillamyvirus*	*Lillamyvirus*	*Pahexavirus*	*Toutatisvirus*
	*Muminvirus*	*Kalppathivirus*	*Muminvirus*	*Brigitvirus*
	*Inovirus*	*Inovirus*	*Lillamyvirus*	*Oengusvirus*
	*Obolenskvirus*	*Anaposvirus*	*Cytomegalovirus*	*Taranisvirus*
	*Pandoravirus*	*Muminvirus*	*Pandoravirus*	*Skunavirus*
Species	*Clostridium phage phiCT453A*	*Clostridium phage phiCT453A*	*Clostridium phage phiCT453A*	*Faecalibacterium_virus_Toutatis*
	*Clostridium phage phiCT9441A*	*Curvibacter virus P26059B*	*Human betaherpesvirus 5*	*Faecalibacterium_virus_Brigit*
	*Escherichia virus M13*	*Escherichia virus M13*	*Flavobacterium virus Filifjonk*	*Faecalibacterium_virus_Oengus*
	*Human betaherpesvirus 5*	*Synechococcus virus SCAM1*	*Clostridium phage phiCT9441A*	*uncultured_crAssphage*
	*Escherichia virus T4*	*Clostridium phage phiCT9441A*	*Lactococcus phage P335 sensulato*	*Faecalibacterium_virus_Taranis*

As shown in **Figure 4B**, in terms of archaea, 19 species were shared by small intestine, cecum and rectum, 2 species were the peculiar species of small intestine. The top 3 phyla of small intestine, cecum and rectum were roughly the same, mainly included Euryarchaeota, Thaumarchaeota and Crenarchaeota. At the genus level, *Halorubrum*, *Methanosarcina* and *Methanobrevibacter* were the top 3 of small intestine, *Halorubrum*, *Methanosarcina* and *Thermococcus* were the top 3 of rectum, only *Halovivax* And *Methanobacterium* were detected in cecum. At the specie level, *Methanobrevibacter smithii*, *Salinadaptatus halalkaliphilus* and *Salinigranum rubrum* were the top 3 of small intestine, Salinadaptatus halalkaliphilus, *Halopiger xanaduensis* and *Methanobrevibacter smithii* were the top 3 of rectum, *Halovivax ruber* was the only specie detected in cecum (**Table 3**).

**Table 3:** Top 5 archaea at phylum, genus and species level (virome).

Level	Small intestine	Cecum	Rectum	Adult gut
Phylum	Euryarchaeota	Euryarchaeota	Euryarchaeota	Euryarchaeota
	Thaumarchaeota	Crenarchaeota	Thaumarchaeota	Crenarchaeota
	Crenarchaeota	Thaumarchaeota	Crenarchaeota	Candidatus Thermoplasmatota
	Candidatus Thermoplasmatota		Candidatus Thermoplasmatota	Thaumarchaeota
	Candidatus Lokiarchaeota		Candidatus Lokiarchaeota	Candidatus_Lokiarchaeota
Genus	*Halorubrum*	*Halovivax*	*Halorubrum*	*Methanosarcina*
	*Methanosarcina*	*Methanobacterium*	*Methanosarcina*	*Methanobrevibacter*
	*Methanobrevibacter*	*Methanobrevibacter*	*Thermococcus*	*Thermococcus*
	*Natrinema*	*Natronococcus*	*Methanobrevibacter*	*Methanococcus*
	*Methanobacterium*	*Halorubrum*	*Methanobacterium*	*Methanosalsum*
Species	*Methanobrevibacter smithii*	*Halovivax ruber*	*Salinadaptatus halalkaliphilus*	*Methanobrevibacter_smithii*
	*Salinadaptatus halalkaliphilus*	*Methanobacterium congolense*	*Halopiger xanaduensis*	*Methanosalsum_zhilinae*
	*Salinigranum rubrum*	*Natronococcus occultus*	*Methanobrevibacter smithii*	*Methanococcus_maripaludis*
	*Halopiger xanaduensis*	*Methanobrevibacter millerae*	*Methanothrix soehngenii*	*Methanosarcina_barkeri*
	*Salinarchaeum sp. Harcht-Bsk1*	*Metallosphaera hakonensis*	*Haloterrigena turkmenica*	*Methanocorpusculum_labreanum*

In terms of bacteria, 1,963 species were shared by small intestine, cecum and rectum, 21 species were the peculiar species of small intestine (**Figure 4C**). Proteobacteria, Actinobacteria and Firmicutes were the predominant phyla in three different parts of fetal gut. The top 3 genera in relative abundances of small intestine and rectum were *Ralstonia*, *Pseudomonas* and *Bradyrhizobium*. *Pseudomonas*, *Ralstonia* and *Mesorhizobium* were the top 3 of cecum. At the specie level, the top 3 of small intestine, cecum and rectum were all composed of *Ralstonia pickettii*, *Cutibacterium acnes* and *Mesorhizobium terrae* (**Table 4**).

**Table 4:** Top 5 bacteria at phylum, genus and species level (virome).

Level	Small intestine	Cecum	Rectum
Phylum	Proteobacteria	Proteobacteria	Proteobacteria
	Actinobacteria	Actinobacteria	Actinobacteria
	Firmicutes	Firmicutes	Firmicutes
	Bacteroidetes	Bacteroidetes	Bacteroidetes
	Planctomycetes	Planctomycetes	Planctomycetes
Genus	*Ralstonia*	*Pseudomonas*	*Pseudomonas*
	*Pseudomonas*	*Ralstonia*	*Ralstonia*
	*Bradyrhizobium*	*Mesorhizobium*	*Bradyrhizobium*
	*Mesorhizobium*	*Bradyrhizobium*	*Mesorhizobium*
	*Cutibacterium*	*Sphingomonas*	*Corynebacterium*
Species	*Ralstonia pickettii*	*Ralstonia pickettii*	*Ralstonia pickettii*
	*Cutibacterium acnes*	*Mesorhizobium terrae*	*Cutibacterium acnes*
	*Mesorhizobium terrae*	*Cutibacterium acnes*	*Mesorhizobium terrae*
	*Bradyrhizobiaceae bacterium SG-6C*	*Methylorubrum extorquens*	*Corynebacterium jeikeium*
	*Clostridium botulinum*	*Methylotenera versatilis*	*Bradyrhizobiaceae bacterium SG-6C*

**Figure 5:**
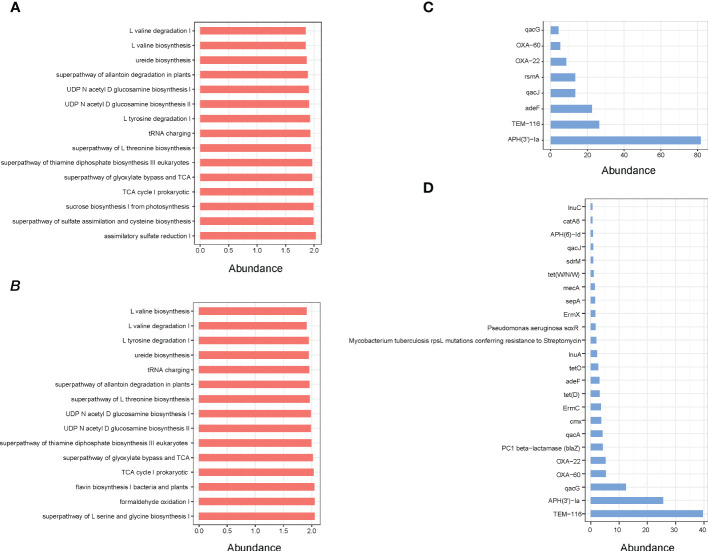
**(A)** Pathway enrichment analyses of small intestine microbiota. **(B)** Pathway enrichment analyses of rectum microbiota. **(C)** ARGs analysis of small intestine microbial. **(D)** ARGs analysis of rectum microbial.

Indeed, the results of functional enrichment analysis showed that the microbes of small intestine and rectum are mainly enriched in the synthesis and metabolism pathways of amino acids and energy metabolism pathways, such as L-valine biosynthesis, ureide biosynthesis, superpathway of glyoxylate bypass and TCA, L-tyrosine degradation I and TCA cycle I prokaryotic (**Figures 5A, B**). Moreover, the identification results of antibiotic resistance genes (ARGs) showed that a total of 25 ARGs were detected, including 8 in small intestine and 24 in rectum, while no ARG was detected in cecum (**Figures 5C, D**).

### The gut microbial composition of adults

**Figure 6:**
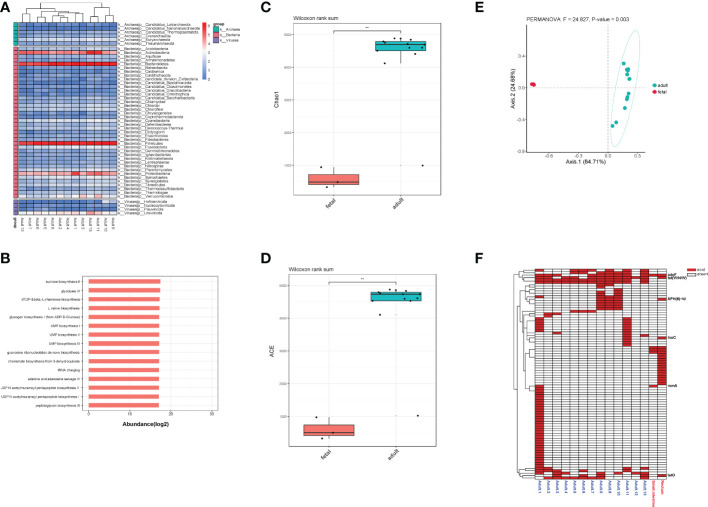
**(A)** The distribution of bacteria, archaea and viruses in each adult sample at phylum level. **(B)** Pathway enrichment analyses of adults’ gut microbiota. **(C)**. Alpha diversity (Chao1 index) estimates between fetal and adult groups. **(D)** Alpha diversity (ACE index) estimates between fetal and adult groups. **(E)** The PCoA plot based on Bray-Curtis distance. **(F)** The distribution of all ARGs in fetal and adult groups. ** means p<0.05, and we chose p value cutoff of 0.05 as the significance level.

For adults’ microbiota, a total of 5385 species of bacteria (40 phyla and 1471 genera), 47 species of viruses (4 phyla and 20 genera) and 258 species of archaea were detected (6 phyla and 112 genera, **Figure 6A**). As shown in **Table 1**, the top 3 phyla were Firmicutes, Bacteroidetes and Actinobacteria. At the genus level, the top 3 were *Bacteroides*, *Phocaeicola* and *Faecalibacterium*. The top 3 species were *Phocaeicola vulgatus*, *Faecalibacterium prausnitzii* and *Bacteroides uniformi*. Since very tiny amounts of viruses and archaea were detected in fetal metagenomic sequencing, we compared the archaea and viruses detected in adults’ metagenomic sequencing to the virome data. In terms of viruses, the top 3 phyla were Uroviricota, Hofneiviricota and Nucleocytoviricota. The top 3 genus were *Toutatisvirus*, *Brigitvirus* and *Oengusvirus*. At the species level, the top 3 were *Faecalibacterium* virus Toutatis, *Faecalibacterium* virus Brigit and *Faecalibacterium* virus Oengus (**Table 2**). As shown in **Table 3**, Euryarchaeota, Crenarchaeota and Candidatus Thermoplasmatota were the top 3 phyla. *Methanosarcina*, *Methanobrevibacter* and *Thermococcus* were the top 3 genera in relative abundances of adults’ microbiota. The top 3 species were *Methanobrevibacter smithii*, *Methanosalsum zhilinae* and *Methanococcus maripaludis*. Moreover, we characterized the global function of adults’ gut microbiota by using HUMANn3. As shown in the **Figure 6B**, the metabolic pathways of adults’ gut microbiota were mainly enriched in sucrose biosynthesis II, glycolysis IV, dTDP &beta; L-rhamnose biosynthesis and L-valine biosynthesis pathways.

To further explore the differences in the microbiota diversity and composition of adults and the fetus, we performed α-diversity and β-diversity analysis. Our results show that there was significant difference in Chao1 and ACE indexes (p<0.05, **Figures 6C, D**). As the principal co-ordinates analysis (PCoA) shown, fetal samples and adult samples were significant separated (p<0.05, **Figure 6E**). Besides, total 71 ARGs were detected in adults’ gut microbiota. Among them, rsmA, tet(W/N/W), adeF, tetO, lnuC and APH(6)-Id were shared by the fetus and adults (**Figure 6F**).

## Discussion

The presence of microbes in fetal gut has long been controversial. After strict experimental conditions and environmental control settings, our results showed that there were indeed microbes in fetal gut, including bacteria, archaea and viruses.

The integration results of metagenomics and virome showed that Proteobacteria, Actinobacteria, Firmicutes and Bacteroidetes were the dominant phyla of fetal gut at the phylum level. At the genus level, *Pseudomonas*, *Bradyrhizobium*, *Microbacterium*, *Burkholderia* and *Ralstonia* were the dominant genera of fetal gut, of which *Ralstonia* and *Burkholderia* were detected in the environmental control groups in recent studies (26). However, *Ralstonia insidiosa*, as a member of *Ralstonia*, was a resident at the maternal-fetal interface in another research (45). Indeed, we also detected a high abundance of *Ralstonia pickettii* and *Ralstonia insidiosa* in fetal gut. Our results confirm that a recent study by Mishra et al. analyzed fetal microbes and found that *Pseudomonas* and *Bradyrhizobium* were enriched in fetal samples (26).

Besides, we detected 700 species viruses, which together with other gut microbial communities maintain the dynamic balance of gut and are key players in the regulation of intestinal homeostasis and inflammation, including 130 species of bacterial viruses (bacteriophages) and 570 species of eukaryotic viruses in fetal gut. (46, 47). Among the 570 species of eukaryotic viruses, *Human betaherpesvirus 5* (also termed human cytomegalovirus) was the most abundant, which is a common cause of congenital viral infection in fetuses and neonates and a major non-genetic cause of congenital sensorineural hearing loss and neurological disability (48, 49). Humans are the only host of human cytomegalovirus (HCMV), which can replicate in most types of cells. HCMV can lead to infection in the developing fetus through vertical transmission during maternal infection (50, 51), and the transmission rate in the first, second, and third trimesters are 26%, 28%, and 65% (52–55). Among the 198 species of bacteriophages that can interact with bacteria to regulate bacterial composition, phages of *Clostridium*, *Escherichia* and *Flavobacterium* were the predominant species. Moreover, we detected crAssphage, which is not only the most abundant virus known to exist in humans but also almost ubiquitous (56), suggesting that crAssphage was acquired in early life. This was contrary to the research from Lim et al. (57).

Most studies currently focused on bacteria, fungi or virus, while archaea are often overlooked. However, the interaction between archaea and host can affect the host in many ways, as archaea were proposed to use for the prevention of trimethylaminuria and cardiovascular disease (58); archaea found on human skin may be related to age and skin physiology (59); archaea participate in the pro-inflammatory process (60). Methane-producing archaea were the predominant component of the archaeome, including Methanobacteriales and Methanomassiliicoccales (61). *Methanobrevibacter smithii* is the most abundant methanogen in the human gut and was isolated as the first representative nearly 40 years ago (62). Take the advantage of virome sequencing, 262 archaeal species (including 8 phyla and 117 genera) were detected in fetal gut. Euryarchaeota was the most abundant phylum, which contained most of the species of archaea (Methanogens, halophiles and Thermophiles). *Methanobrevibacter smithii* was the most abundant specie, and this is the first ever detection in fetal gut of *Methanobrevibacter smithii*, which confirmed the hypothesis from Sereme et al. that *Methanobrevibacter smithii* was an *in-utero* member of gut microbiota (63). Our results opposed the hypothesis that breast milk is the source of *Methanobrevibacter smithii* in premature neonates (64). The study from Grine et al. showed that *Methanobrevibacter smithii* was detected in vaginal fluid only in cases of vaginal disease (65), although the fetus passed through the vagina, the mother of our study without bacterial vaginosis. Besides, more methanogens such as *Methanobrevibacter millerae*, *Methanobrevibacter olleyae*, *Methanobrevibacter ruminantium* and *Methanosphaera stadtmanae* were detected in fetal gut.

Both metagenomic and virome sequencing results showed that rectal microbial species were higher than those of small intestine and cecum in different classification levels (phylum, genus and specie). Besides, the composition and functional pathway of the small intestine and rectum were more similar. The difference between the cecum and the other two sites might be due to the host contamination of the cecum was more severe than that of the small intestine and rectum.

We compared the fetal gut microbiota to the adults’ and found that the diversity of adults’ gut microbiota was significantly higher than that in fetal group. The composition of gut microbiota between two groups was significantly different. Specifically, Proteobacteria and Actinobacteria were the dominant bacteria phyla in fetal gut. While the dominant bacteria phyla in adults’ gut were Firmicutes and Bacteroidetes, which indicated that Firmicutes and Bacteroidetes gradually replaced Proteobacteria and Actinobacteria as the main force of gut microbiota during the process of growth and development. For viruses and archaea, the dominant phyla in both groups were similar. Besides, we found 6 ARGs in fetal group were consistent with adult group, suggesting that microbes carried ARGs might transmit vertically during pregnancy.

In conclusion, we detected a variety of microbes in fetal gut through metagenomic and virome sequencing, including bacteria, virus and archaea. Especially, we first identified *Methanobrevibacter smithii* in fetal gut, which was the most prevalent and abundant methanogen. In addition, by comparing the fetal and adults’ gut microbiota, we found that gut bacterial composition changed greatly during the growth and development process and the same ARGs existed in fetuses and adults. Thus, we suggest the fetal gut is not a sterile environment and has a low abundance but metabolically rich microbiome. Our study provided valuable resource for understanding human fetal immunity development during gestation.

## Data availability statement

The data presented in the study are deposited in the Genome Sequence Archive for Human repository, accession number: HRA003676.

## Ethics statement

The studies involving human participants were reviewed and approved by West china 2nd university hospital. The patients/participants provided their written informed consent to participate in this study. Written informed consent was obtained from the individual(s), and minor(s)’ legal guardian/next of kin, for the publication of any potentially identifiable images or data included in this article.

## Author contributions

Experimental work: GH, AL, JL and MH. Data analysis: YL, XYL and WG. Writing and editing: XL, XHL, ZF and YZ. All authors contributed to the article and approved the submitted version.

## References

[1] Tieppo P Papadopoulou M Gatti D Mcgovern N Chan JKY Gosselin F. The human fetal thymus generates invariant effector γδ T cells. J Exp Med (2019) 217:e20190580. doi: 10.1084/jem.20190580 PMC7062527 31816633

[2] Park JE Botting RA Domínguez Conde C Popescu DM Lavaert M Kunz DJ. A cell atlas of human thymic development defines T cell repertoire formation. Science (2020) 367. doi: 10.1126/science.aay3224 PMC7611066 32079746

[3] Rackaityte E Halkias J. Mechanisms of fetal T cell tolerance and immune regulation. Front Immunol (2020) 11:588. doi: 10.3389/fimmu.2020.00588 32328065 PMC7160249

[4] Jain N. The early life education of the immune system: Moms, microbes and (missed) opportunities. Gut Microbes (2020) 12:1824564. doi: 10.1080/19490976.2020.1824564 33043833 PMC7781677

[5] Digiulio DB Gervasi M Romero R Mazaki-Tovi S Vaisbuch E Kusanovic JP. Microbial invasion of the amniotic cavity in preeclampsia as assessed by cultivation and sequence-based methods. J Perinat Med (2010) 38:503–13. doi: 10.1515/jpm.2010.078 PMC3325506 20482470

[6] Gervasi MT Romero R Bracalente G Chaiworapongsa T Erez O Dong Z. Viral invasion of the amniotic cavity (VIAC) in the midtrimester of pregnancy. J Matern Fetal Neonatal Med (2012) 25:2002–13. doi: 10.3109/14767058.2012.683899 PMC3498469 22524157

[7] Romero R Miranda J Chaiworapongsa T Chaemsaithong P Gotsch F Dong Z. A novel molecular microbiologic technique for the rapid diagnosis of microbial invasion of the amniotic cavity and intra-amniotic infection in preterm labor with intact membranes. Am J Reprod Immunol (2014) 71:330–58. doi: 10.1111/aji.12189 PMC3954440 24417618

[8] Romero R Miranda J Chaemsaithong P Chaiworapongsa T Kusanovic JP Dong Z. Sterile and microbial-associated intra-amniotic inflammation in preterm prelabor rupture of membranes. J Matern Fetal Neonatal Med (2015) 28:1394–409. doi: 10.3109/14767058.2014.958463 PMC5371030 25190175

[9] Shin H Pei Z Martinez KA 2nd Rivera-Vinas JI Mendez K Cavallin H. The first microbial environment of infants born by c-section: the operating room microbes. Microbiome (2015) 3:59. doi: 10.1186/s40168-015-0126-1 26620712 PMC4665759

[10] Lauder AP Roche AM Sherrill-Mix S Bailey A Laughlin AL Bittinger K. Comparison of placenta samples with contamination controls does not provide evidence for a distinct placenta microbiota. Microbiome (2016) 4:29. doi: 10.1186/s40168-016-0172-3 27338728 PMC4917942

[11] Arora N Sadovsky Y Dermody TS Coyne CB. Microbial vertical transmission during human pregnancy. Cell Host Microbe (2017) 21:561–7. doi: 10.1016/j.chom.2017.04.007 PMC6148370 28494237

[12] Maidji E Nigro G Tabata T Mcdonagh S Nozawa N Shiboski S. Antibody treatment promotes compensation for human cytomegalovirus-induced pathogenesis and a hypoxia-like condition in placentas with congenital infection. Am J Pathol (2010) 177:1298–310. doi: 10.2353/ajpath.2010.091210 PMC2928963 20651234

[13] Robbins JR Skrzypczynska KM Zeldovich VB Kapidzic M Bakardjiev AI. Placental syncytiotrophoblast constitutes a major barrier to vertical transmission of listeria monocytogenes. PloS Pathog (2010) 6:e1000732. doi: 10.1371/journal.ppat.1000732 20107601 PMC2809766

[14] Delorme-Axford E Donker RB Mouillet JF Chu T Bayer A Ouyang Y. Human placental trophoblasts confer viral resistance to recipient cells. Proc Natl Acad Sci U.S.A. (2013) 110:12048–53. doi: 10.1073/PNAS.1304718110 PMC3718097 23818581

[15] Bayer A Delorme-Axford E Sleigher C Frey TK Trobaugh DW Klimstra WB. Human trophoblasts confer resistance to viruses implicated in perinatal infection. Am J Obstet Gynecol (2015) 212:71.e71–8. doi: 10.1016/j.ajog.2014.07.060 PMC4275367 25108145

[16] Bayer A Lennemann NJ Ouyang Y Bramley JC Morosky S Marques ET Jr.. Type III interferons produced by human placental trophoblasts confer protection against zika virus infection. Cell Host Microbe (2016) 19:705–12. doi: 10.1016/j.chom.2016.03.008 PMC4866896 27066743

[17] Diaz Heijtz R. Fetal, neonatal, and infant microbiome: Perturbations and subsequent effects on brain development and behavior. Semin Fetal Neonatal Med (2016) 21:410–7. doi: 10.1016/j.siny.2016.04.012 27255860

[18] Kundu P Blacher E Elinav E Pettersson S. Our gut microbiome: The evolving inner self. Cell (2017) 171:1481–93. doi: 10.1016/j.cell.2017.11.024 29245010

[19] Perez-Muñoz ME Arrieta MC Ramer-Tait AE Walter J. A critical assessment of the "sterile womb" and "*in utero* colonization" hypotheses: implications for research on the pioneer infant microbiome. Microbiome (2017) 5:48. doi: 10.1186/s40168-017-0268-4 28454555 PMC5410102

[20] Willyard C. Could baby's first bacteria take root before birth? Nature (2018) 553:264–6. doi: 10.1038/d41586-018-00664-8 29345664

[21] De Goffau MC Lager S Sovio U Gaccioli F Cook E Peacock SJ. Human placenta has no microbiome but can contain potential pathogens. Nature (2019) 572:329–34. doi: 10.1038/s41586-019-1451-5 PMC6697540 31367035

[22] Seferovic MD Pace RM Carroll M Belfort B Major AM Chu DM. Visualization of microbes by 16S *in situ* hybridization in term and preterm placentas without intraamniotic infection. Am J Obstet Gynecol (2019) 221:146.e141–146.e123. doi: 10.1016/j.ajog.2019.04.036 PMC10357491 31055031

[23] Younge N Mccann JR Ballard J Plunkett C Akhtar S Araújo-Pérez F. Fetal exposure to the maternal microbiota in humans and mice. JCI Insight (2019) 4. doi: 10.1172/jci.insight.127806 PMC6795398 31479427

[24] Al Alam D Danopoulos S Grubbs B Ali N Macaogain M Chotirmall SH. Human fetal lungs harbor a microbiome signature. Am J Respir Crit Care Med (2020) 201:1002–6. doi: 10.1164/rccm.201911-2127LE PMC7159424 31898918

[25] Rackaityte E Halkias J Fukui EM Mendoza VF Hayzelden C Crawford ED. Viable bacterial colonization is highly limited in the human intestine *in utero* . Nat Med (2020) 26:599–607. doi: 10.1038/s41591-020-0761-3 32094926 PMC8110246

[26] Mishra A Lai GC Yao LJ Aung TT Shental N Rotter-Maskowitz A. Microbial exposure during early human development primes fetal immune cells. Cell (2021) 184:3394–3409.e3320. doi: 10.1016/j.cell.2021.04.039 34077752 PMC8240556

[27] Sterpu I Fransson E Hugerth LW Du J Pereira M Cheng L. No evidence for a placental microbiome in human pregnancies at term. Am J Obstet Gynecol (2021) 224:296.e291–296.e223. doi: 10.1016/j.ajog.2020.08.103 32871131

[28] Aagaard K Ma J Antony KM Ganu R Petrosino J Versalovic J. The placenta harbors a unique microbiome. Sci Transl Med (2014) 6:237ra265. doi: 10.1126/scitranslmed.3008599 PMC4929217 24848255

[29] Leon LJ Doyle R Diez-Benavente E Clark TG Klein N Stanier P. Enrichment of clinically relevant organisms in spontaneous preterm-delivered placentas and reagent contamination across all clinical groups in a Large pregnancy cohort in the united kingdom. Appl Environ Microbiol (2018) 84. doi: 10.1128/AEM.00483-18 PMC6029081 29776928

[30] Weisblum Y Panet A Zakay-Rones Z Haimov-Kochman R Goldman-Wohl D Ariel I. Modeling of human cytomegalovirus maternal-fetal transmission in a novel decidual organ culture. J Virol (2011) 85:13204–13. doi: 10.1128/JVI.05749-11 PMC3233115 21976654

[31] Tabata T Petitt M Puerta-Guardo H Michlmayr D Wang C Fang-Hoover J. Zika virus targets different primary human placental cells, suggesting two routes for vertical transmission. Cell Host Microbe (2016) 20:155–66. doi: 10.1016/j.chom.2016.07.002 PMC5257282 27443522

[32] Theis KR Romero R Winters AD Greenberg JM Gomez-Lopez N Alhousseini A. Does the human placenta delivered at term have a microbiota? results of cultivation, quantitative real-time PCR, 16S rRNA gene sequencing, and metagenomics. Am J Obstet Gynecol (2019) 220:267.e261–267.e239. doi: 10.1016/j.ajog.2018.10.018 PMC6733039 30832984

[33] Bi Y Tu Y Zhang N Wang S Zhang F Suen G. Multiomics analysis reveals the presence of a microbiome in the gut of fetal lambs. Gut (2021) 70:853–64. doi: 10.1136/gutjnl-2020-320951 PMC8040156 33589511

[34] Qin J Li R Raes J Arumugam M Burgdorf KS Manichanh C. A human gut microbial gene catalogue established by metagenomic sequencing. Nature (2010) 464:59–65. doi: 10.1038/nature08821 20203603 PMC3779803

[35] Shkoporov AN Clooney AG Sutton TDS Ryan FJ Daly KM Nolan JA. The human gut virome is highly diverse, stable, and individual specific. Cell Host Microbe (2019) 26:527–541.e525. doi: 10.1016/j.chom.2019.09.009 31600503

[36] Polkinghorne DE. Phenomenological research methods. In: Valle RS Halling S, editors. Existential-phenomenological perspectives in psychology: Exploring the breadth of human experience. Boston, MA: Springer US (1989). p. 41–60.

[37] Bolger AM Lohse M Usadel B. Trimmomatic: a flexible trimmer for illumina sequence data. Bioinformatics (2014) 30:2114–20. doi: 10.1093/bioinformatics/btu170 PMC4103590 24695404

[38] Langmead B Salzberg SL. Fast gapped-read alignment with bowtie 2. Nat Methods (2012) 9:357–9. doi: 10.1038/nmeth.1923 PMC3322381 22388286

[39] Li D Liu CM Luo R Sadakane K Lam TW. MEGAHIT: an ultra-fast single-node solution for large and complex metagenomics assembly *via* succinct de bruijn graph. Bioinformatics (2015) 31:1674–6. doi: 10.1093/bioinformatics/btv033 25609793

[40] Hyatt D Chen GL Locascio PF Land ML Larimer FW Hauser LJ. Prodigal: prokaryotic gene recognition and translation initiation site identification. BMC Bioinf (2010) 11:119. doi: 10.1186/1471-2105-11-119 PMC2848648 20211023

[41] Buchfink B Xie C Huson DH. Fast and sensitive protein alignment using DIAMOND. Nat Methods (2015) 12:59–60. doi: 10.1038/nmeth.3176 25402007

[42] Wood DE Salzberg SL. Kraken: ultrafast metagenomic sequence classification using exact alignments. Genome Biol (2014) 15:R46. doi: 10.1186/gb-2014-15-3-r46 24580807 PMC4053813

[43] Franzosa EA Mciver LJ Rahnavard G Thompson LR Schirmer M Weingart G. Species-level functional profiling of metagenomes and metatranscriptomes. Nat Methods (2018) 15:962–8. doi: 10.1038/s41592-018-0176-y PMC6235447 30377376

[44] Alcock BP Raphenya AR Lau TTY Tsang KK Bouchard M Edalatmand A. CARD 2020: antibiotic resistome surveillance with the comprehensive antibiotic resistance database. Nucleic Acids Res (2020) 48:D517–d525. doi: 10.1093/nar/gkz935 31665441 PMC7145624

[45] Parnell LA Willsey GG Joshi CS Yin Y Wargo MJ Mysorekar IU. Functional characterization of ralstonia insidiosa, a bona fide resident at the maternal-fetal interface. bioRxiv (2019), 721977. doi: 10.1101/721977

[46] Lee YK Mazmanian SK. Has the microbiota played a critical role in the evolution of the adaptive immune system? Science (2010) 330:1768–73. doi: 10.1126/science.1195568 PMC3159383 21205662

[47] Norman JM Handley SA Virgin HW. Kingdom-agnostic metagenomics and the importance of complete characterization of enteric microbial communities. Gastroenterology (2014) 146:1459–69. doi: 10.1053/j.gastro.2014.02.001 PMC4009354 24508599

[48] Kenneson A Cannon MJ. Review and meta-analysis of the epidemiology of congenital cytomegalovirus (CMV) infection. Rev Med Virol (2007) 17:253–76. doi: 10.1002/rmv.535 17579921

[49] Wang C Zhang X Bialek S Cannon MJ. Attribution of congenital cytomegalovirus infection to primary versus non-primary maternal infection. Clin Infect Dis (2011) 52:e11–13. doi: 10.1093/cid/ciq085 21288834

[50] Fowler KB Stagno S Pass RF. Maternal immunity and prevention of congenital cytomegalovirus infection. Jama (2003) 289:1008–11. doi: 10.1001/jama.289.8.1008 12597753

[51] Yamamoto AY Mussi-Pinhata MM Boppana SB Novak Z Wagatsuma VM Oliveira Pde F. Human cytomegalovirus reinfection is associated with intrauterine transmission in a highly cytomegalovirus-immune maternal population. Am J Obstet Gynecol (2010) 202:297.e291–298. doi: 10.1016/j.ajog.2009.11.018 PMC8351475 20060091

[52] Bodéus M Hubinont C Goubau P. Increased risk of cytomegalovirus transmission *in utero* during late gestation. Obstet Gynecol (1999) 93:658–60. 10912962

[53] Enders G Daiminger A Bäder U Exler S Enders M. Intrauterine transmission and clinical outcome of 248 pregnancies with primary cytomegalovirus infection in relation to gestational age. J Clin Virol (2011) 52:244–6. doi: 10.1016/j.jcv.2011.07.005 21820954

[54] Feldman B Yinon Y Tepperberg Oikawa M Yoeli R Schiff E Lipitz S. Pregestational, periconceptional, and gestational primary maternal cytomegalovirus infection: prenatal diagnosis in 508 pregnancies. Am J Obstet Gynecol (2011) 205:342.e341–346. doi: 10.1016/j.ajog.2011.05.030 21741614

[55] Leruez-Ville M Foulon I Pass R Ville Y. Cytomegalovirus infection during pregnancy: state of the science. Am J Obstet Gynecol (2020) 223:330–49. doi: 10.1016/j.ajog.2020.02.018 32105678

[56] Dutilh BE Cassman N Mcnair K Sanchez SE Silva GG Boling L. A highly abundant bacteriophage discovered in the unknown sequences of human faecal metagenomes. Nat Commun (2014) 5:4498. doi: 10.1038/ncomms5498 25058116 PMC4111155

[57] Lim ES Zhou Y Zhao G Bauer IK Droit L Ndao IM. Early life dynamics of the human gut virome and bacterial microbiome in infants. Nat Med (2015) 21:1228–34. doi: 10.1038/nm.3950 PMC4710368 26366711

[58] Brugère JF Borrel G Gaci N Tottey W O'toole PW Malpuech-Brugère C. Archaebiotics: proposed therapeutic use of archaea to prevent trimethylaminuria and cardiovascular disease. Gut Microbes (2014) 5:5–10. doi: 10.4161/gmic.26749 24247281 PMC4049937

[59] Probst AJ Auerbach AK Moissl-Eichinger C. Archaea on human skin. PloS One (2013) 8:e65388. doi: 10.1371/journal.pone.0065388 23776475 PMC3680501

[60] Bang C Vierbuchen T Gutsmann T Heine H Schmitz RA. Immunogenic properties of the human gut-associated archaeon methanomassiliicoccus luminyensis and its susceptibility to antimicrobial peptides. PloS One (2017) 12:e0185919. doi: 10.1371/journal.pone.0185919 28982164 PMC5628862

[61] Borrel G Brugère JF Gribaldo S Schmitz RA Moissl-Eichinger C. The host-associated archaeome. Nat Rev Microbiol (2020) 18:622–36. doi: 10.1038/s41579-020-0407-y 32690877

[62] Miller TL Wolin MJ Conway De Macario E Macario AJ. Isolation of methanobrevibacter smithii from human feces. Appl Environ Microbiol (1982) 43:227–32. doi: 10.1128/aem.43.1.227-232.1982 PMC241804 6798932

[63] Sereme Y Guindo CO Filleron A Corbeau P Tran TA Drancourt M. Meconial methanobrevibacter smithii suggests intrauterine methanogen colonization in preterm neonates. Curr Res Microb Sci (2021) 2:100034.doi: 10.1016/j.crmicr.2021.100034 34841325 PMC8610290

[64] Togo AH Grine G Khelaifia S Des Robert C Brevaut V Caputo A. Culture of methanogenic archaea from human colostrum and milk. Sci Rep (2019) 9:18653. doi: 10.1038/s41598-019-54759-x 31819085 PMC6901439

[65] Grine G Drouet H Fenollar F Bretelle F Raoult D Drancourt M. Detection of methanobrevibacter smithii in vaginal samples collected from women diagnosed with bacterial vaginosis. Eur J Clin Microbiol Infect Dis (2019) 38:1643–9. doi: 10.1007/s10096-019-03592-1 31127480

